# Dependence of Nurses’ Professional Burden on the Length of Work in Psychiatric Institutions

**DOI:** 10.1192/j.eurpsy.2026.10973

**Published:** 2026-07-31

**Authors:** V. Mitikhin, T. Solokhina, D. Oshevsky

**Affiliations:** Mental Health Research Centre, Moscow, Russian Federation

## Abstract

**Introduction:**

Modern psychiatric care requires high professional skill and personal stability of medical staff, especially nurses. Cross-cultural and national studies (V. Yastrebov, O. Mitina 2012; Tolosa-Merlos D, et.al., 2023; V. Mitikhin, T. Solokhina, D. Oshevsky, 2024) have revealed that social and demographic characteristics and conditions of work can negatively impact physical and emotional well-being of nurses. Given this, it is important to identify the parameters that influence the formation of the professional burden of nurses and to find measures to reduce it.

**Objectives:**

To assess the dependence of the professional burden on length of work in psychiatric institutions and to develop measures to reduce it.

**Methods:**

The “Questionnaire for assessing the burden of workers in psychiatric institutions” (WHO, 1994) was used. The study involved 502 nurses with an average age of 44,99 ± 11,18 years. The participants were divided into three groups according to the length of work: up to 5 years, from 5 to 10 years and more than 10 years. The indicators of professional burden were presented as median values with interquartile range (Me [Q1; Q3]). The Kruskal-Wallis criterion (H-test) was used to compare the results between groups.

**Results:**

In the overall sample the indicators of professional burden for all questionnaire items were at an average level and were characterized by low variability. However, when comparing the groups by the length of work, differences in the indicators of professional burden were revealed. With an increase in length of work a significant growth in the subjective feeling of deterioration in physical health (р=0,006) was noted: up to 5 years, the indicators were 1,17 [1,17; 1,33], from 5 to 10 years – 1,17 [1,17; 1,58], more than 10 years – 1,33 [1,17; 1,67]. A significant deterioration in the indicators of psychological well-being (р=0,014) was also observed: up to 5 years – 1,17 [1,00; 1,33], from 5 to 10 years – 1,00 [0,43; 1,57], more than 10 years – 1,33 [1,00; 1,50]. Statistically significant differences were identified between the groups with length of work less than 5 years and more than 10 years. Differences in the integrated burden indicator were noted at the trend level. More than 35% of nurses with 5–10 years length of work and nearly half of those with over 10 years have considered seeking alternative employment.

**Conclusions:**

The length of work in psychiatric institutions should be considered when planning strategies to reduce professional burden on the medical staff. These strategies may include arrangement of a comfortable workplace, maintaining balance of work and rest, creating a favorable microclimate. It is also important to introduce psychological support and to improve team methods of work.

**Disclosure of Interest:**

None Declared

